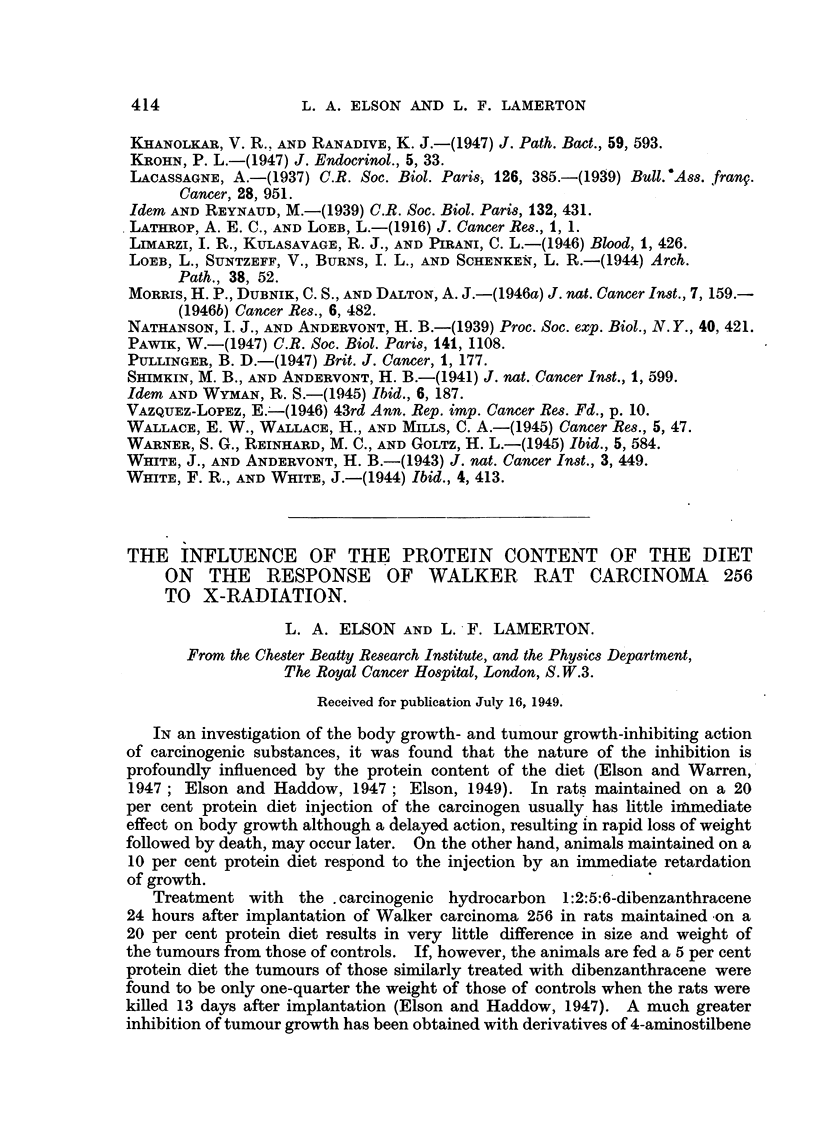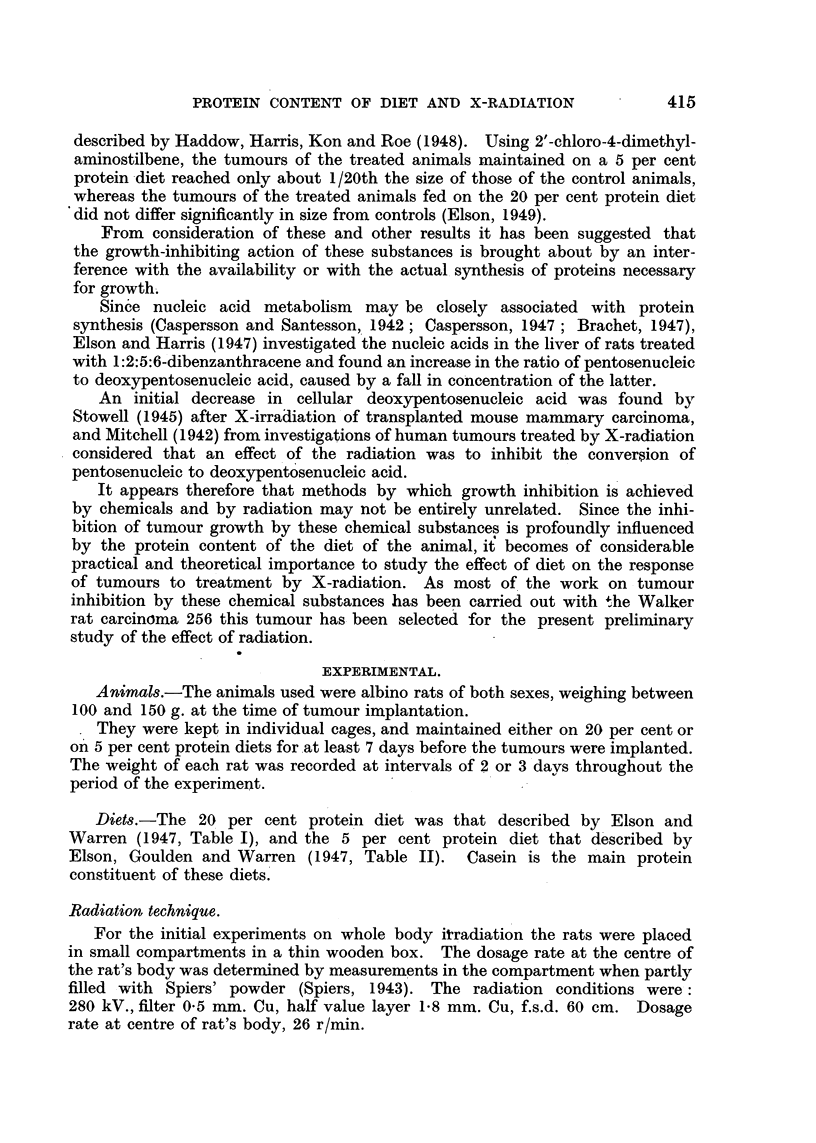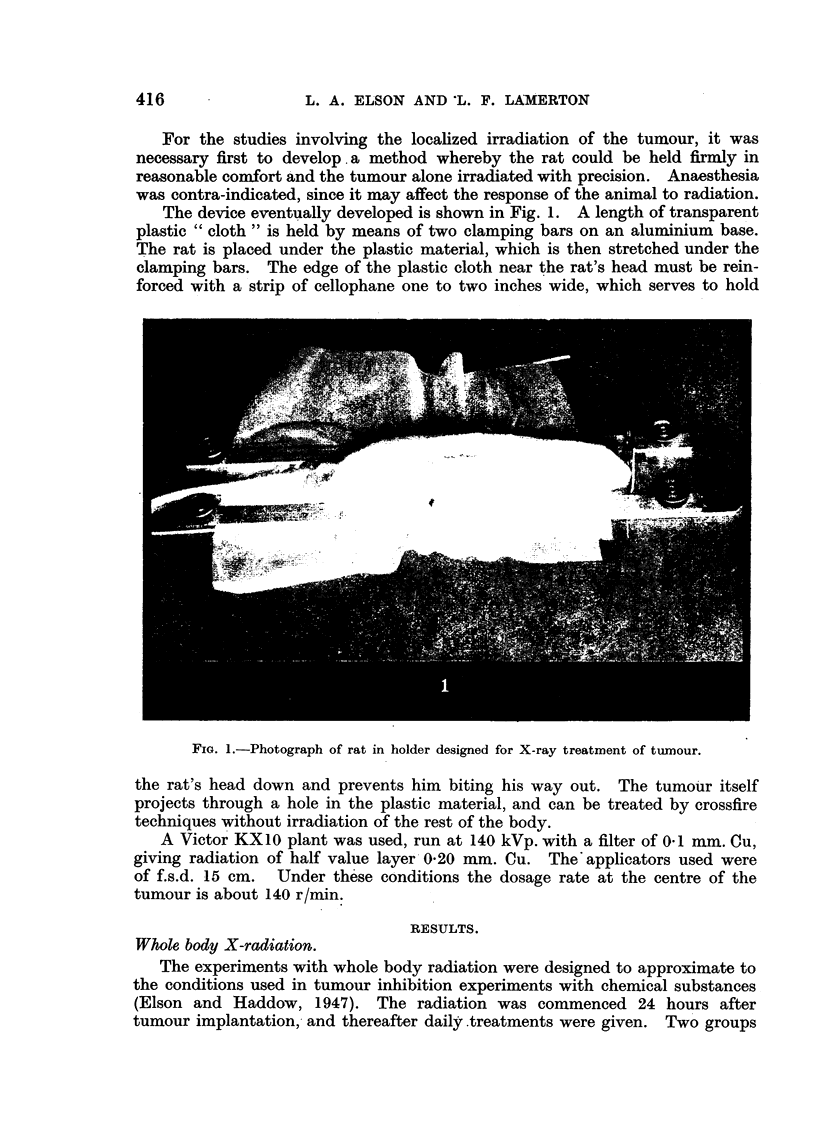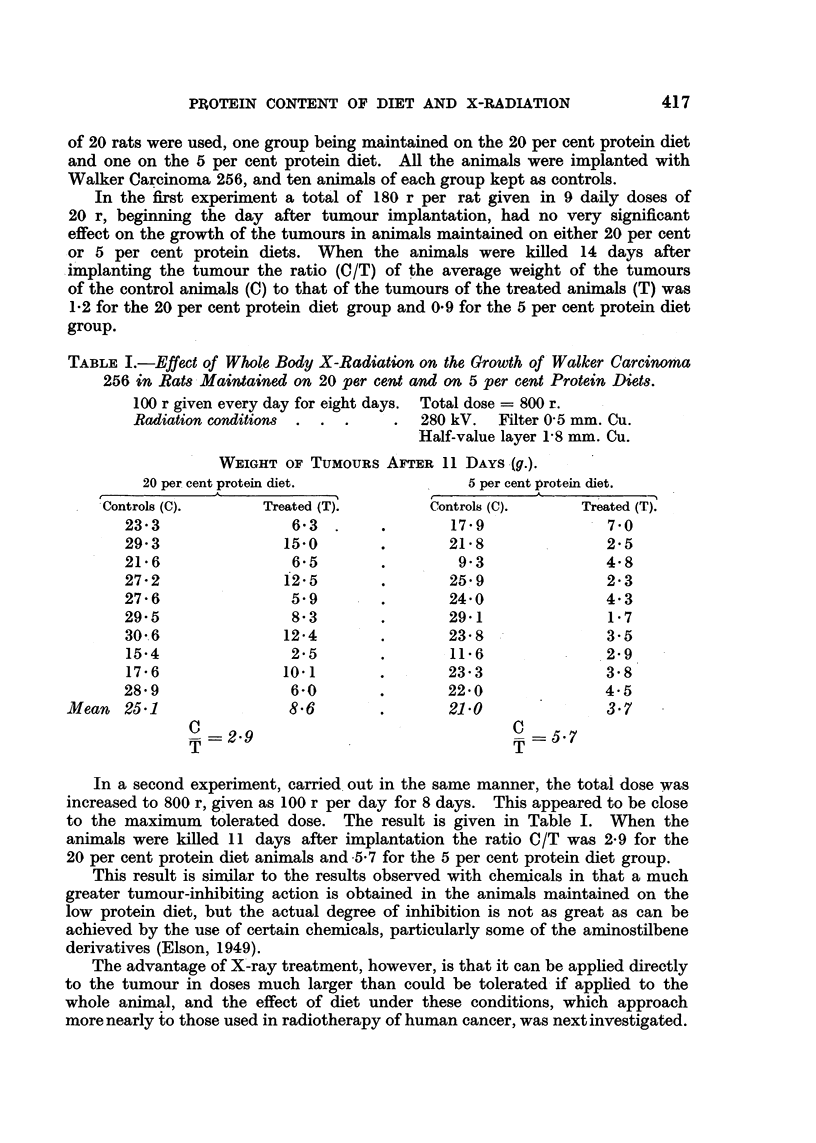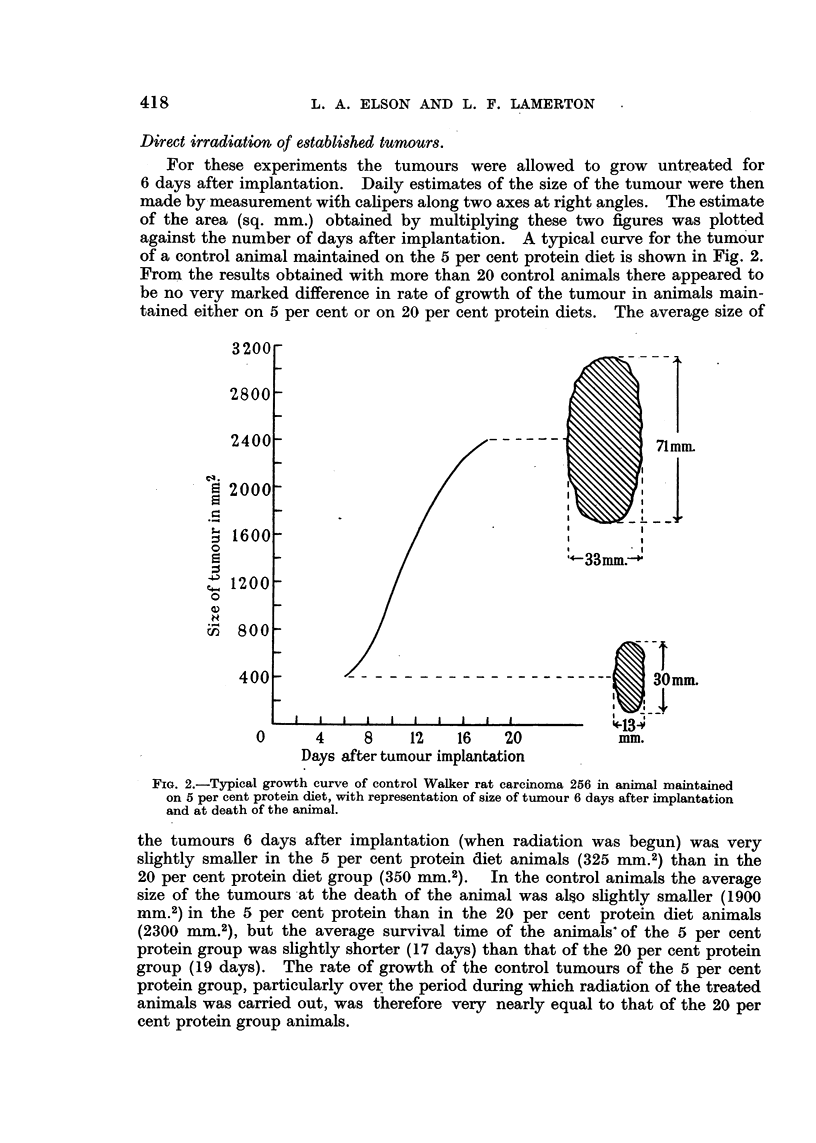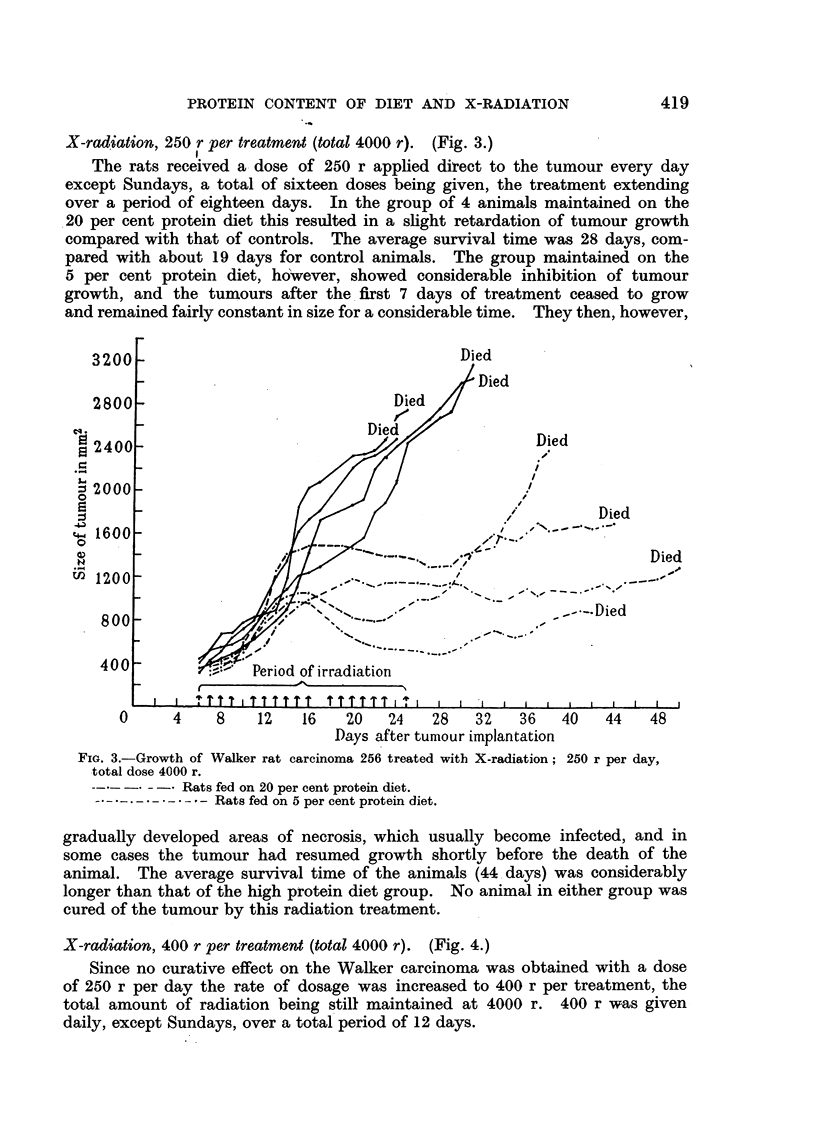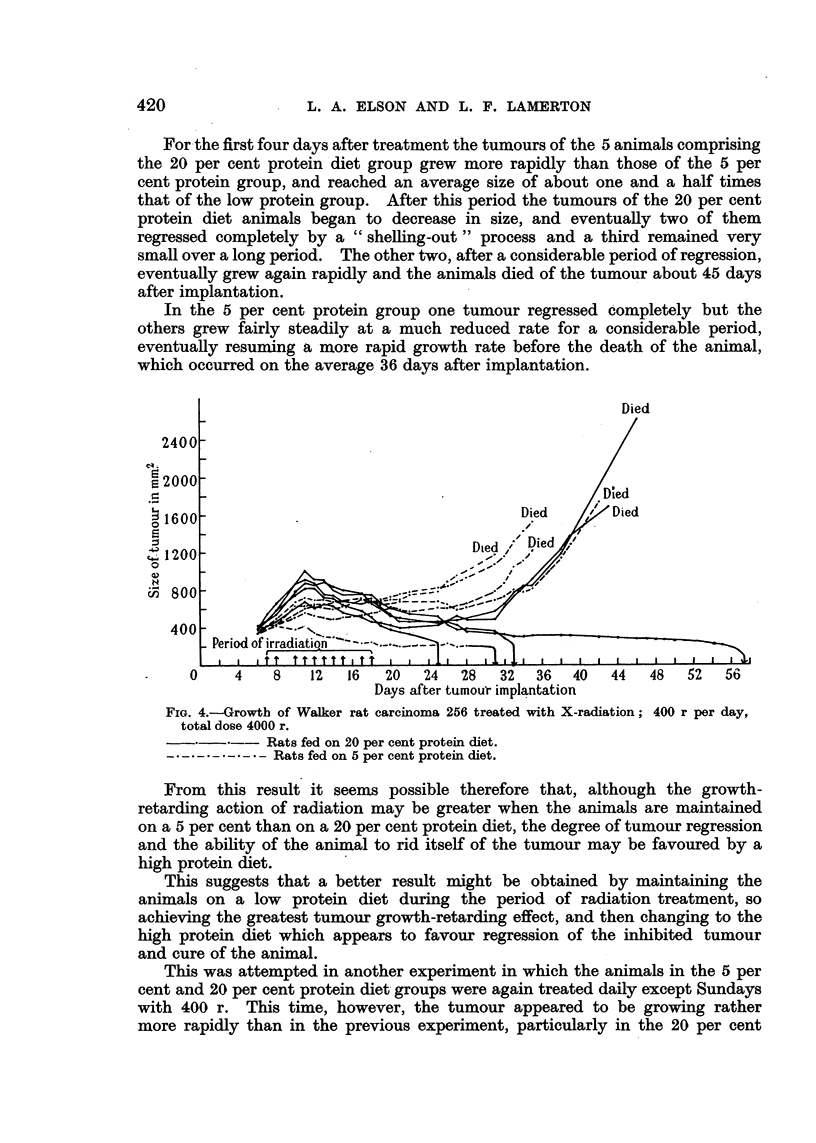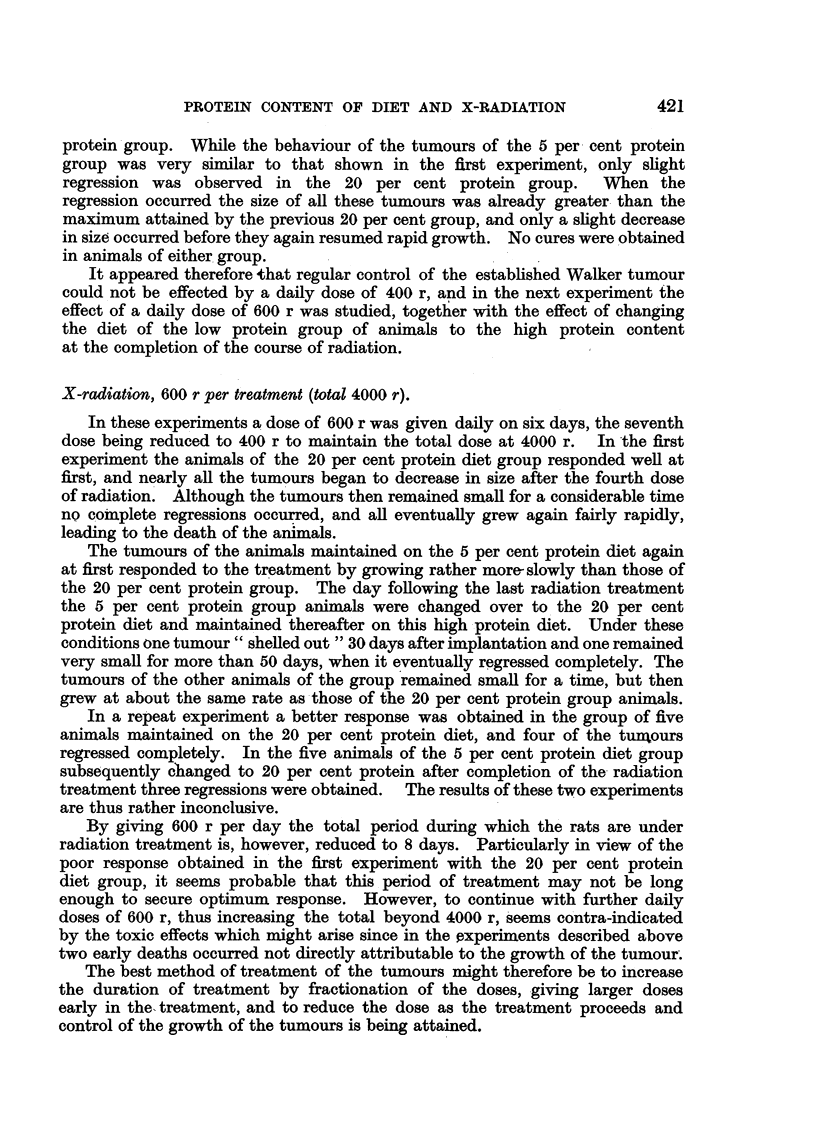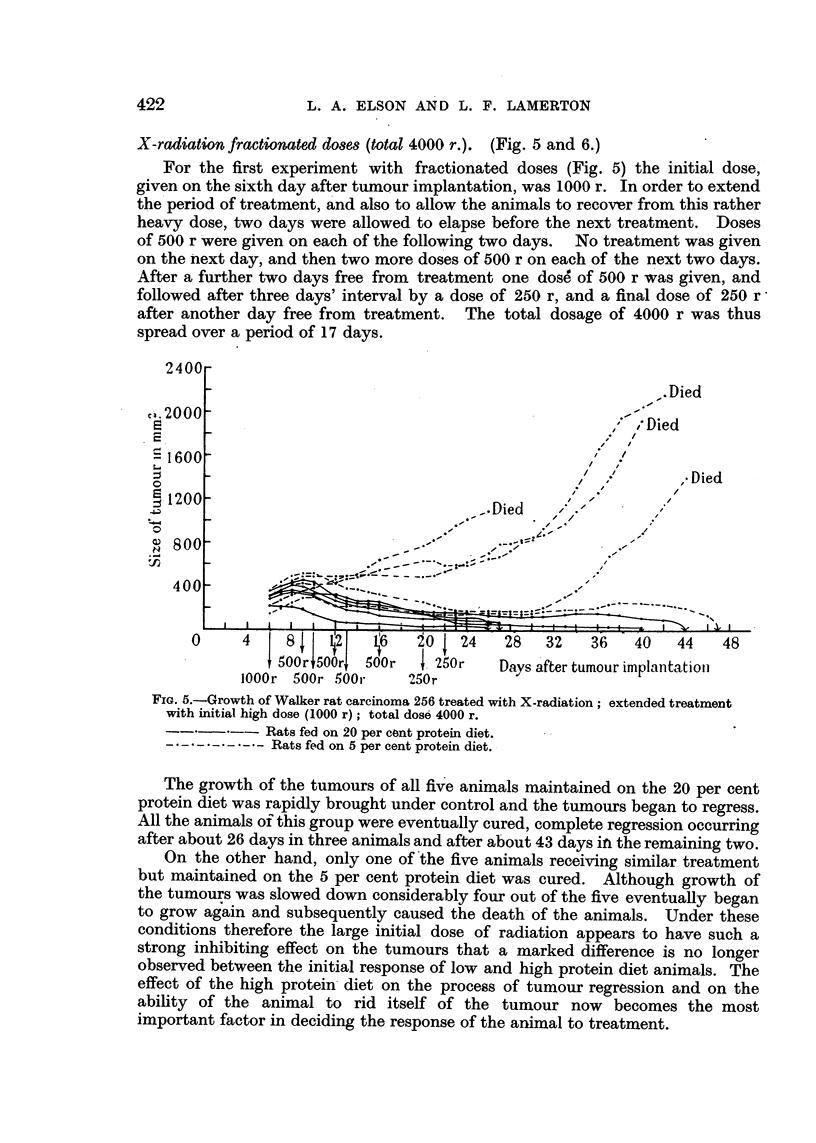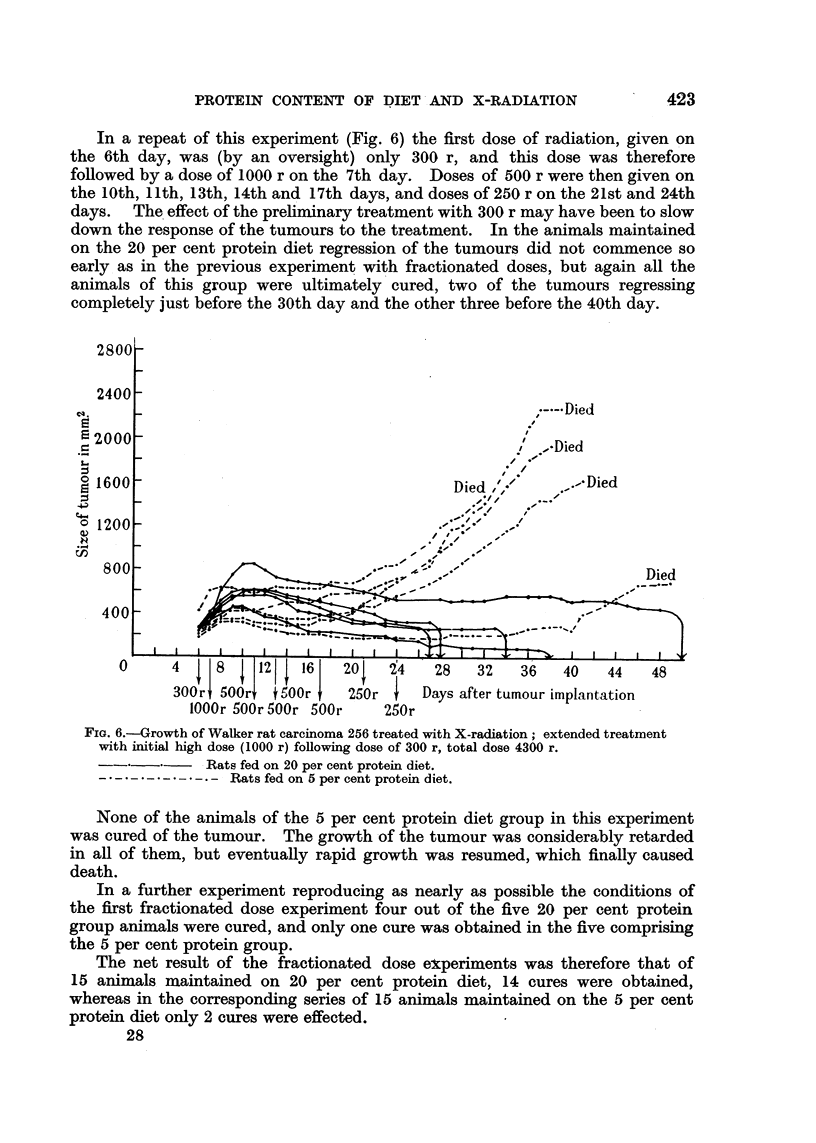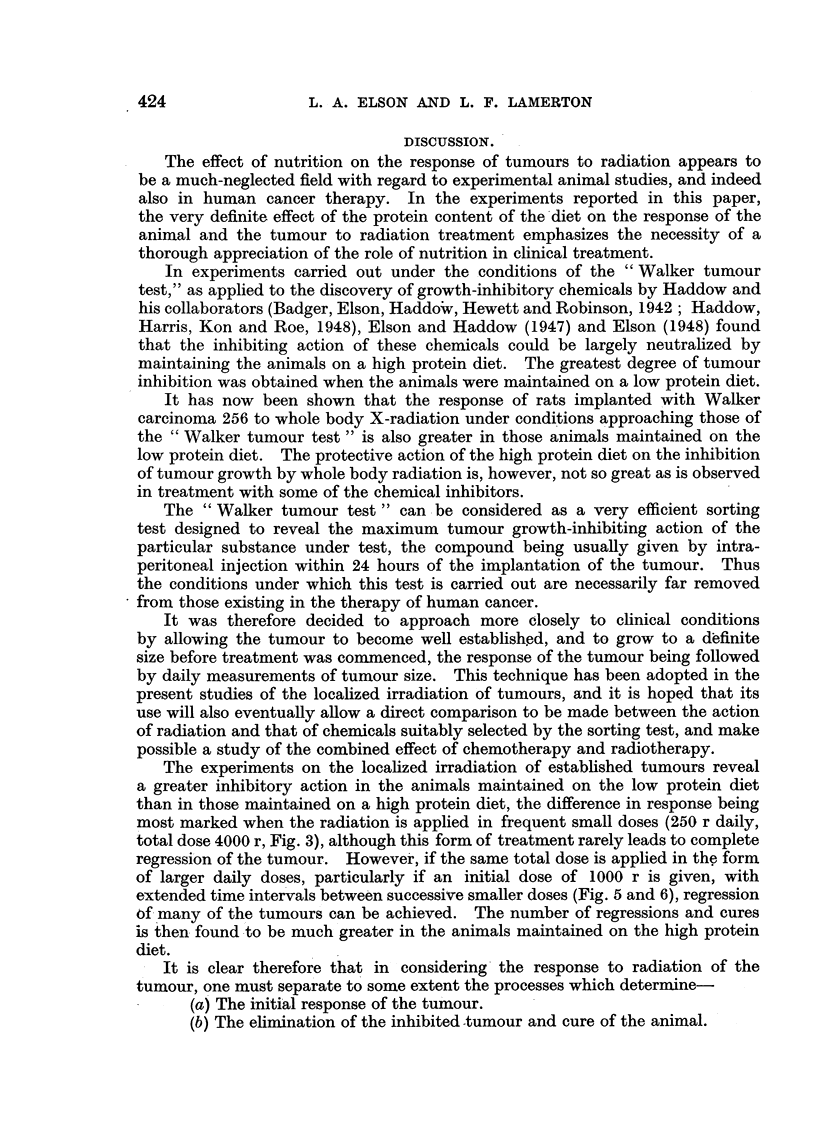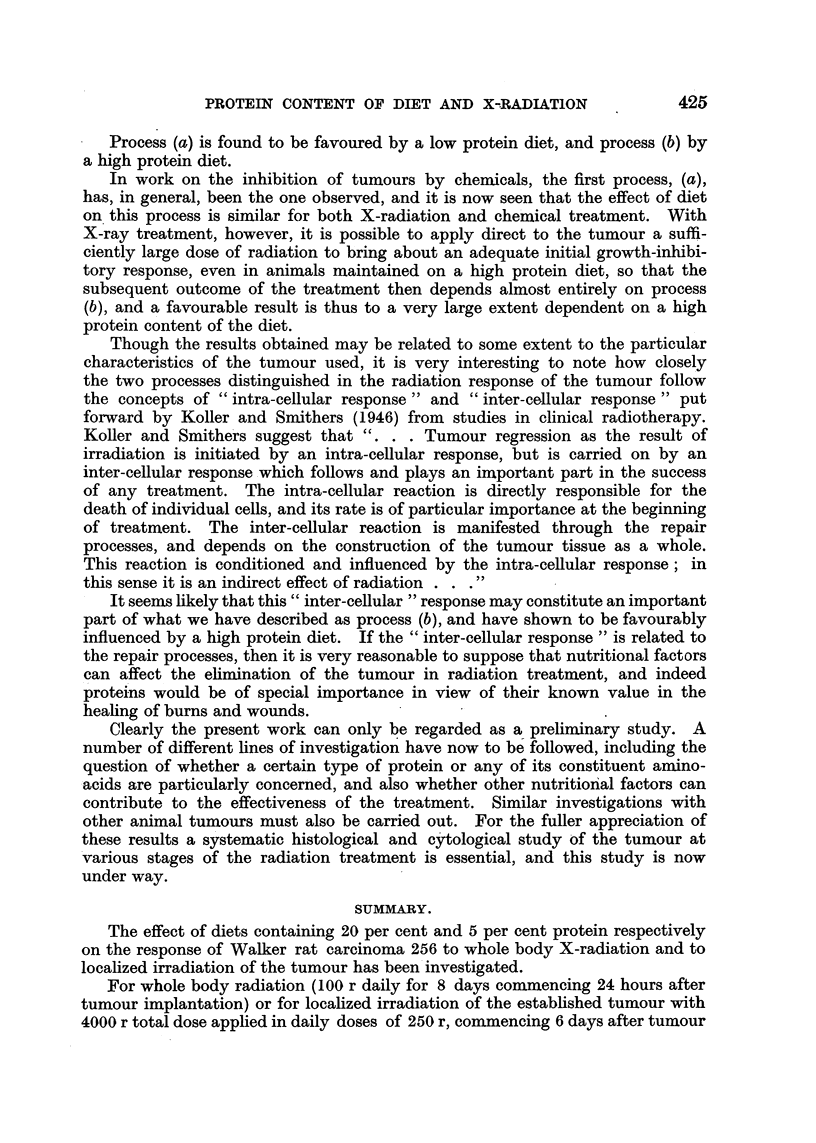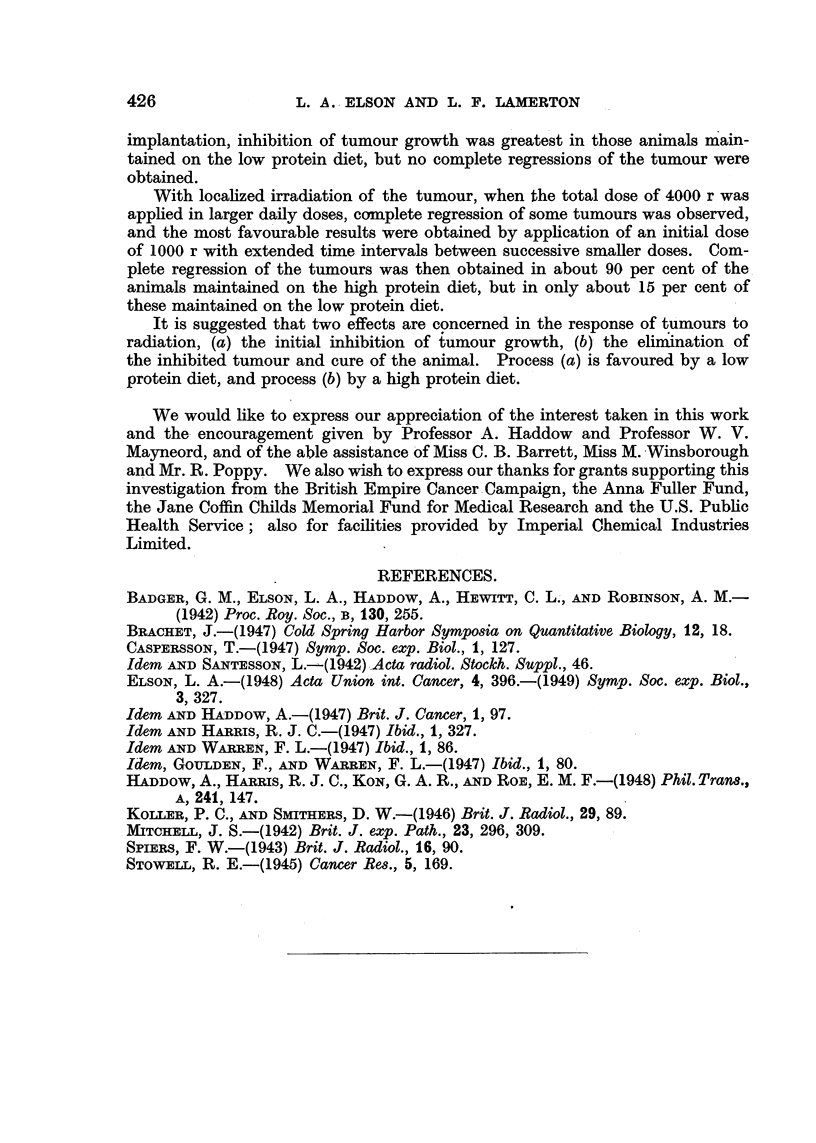# The Influence of the Protein Content of the Diet on the Response of Walker Rat Carcinoma 256 to X-radiation

**DOI:** 10.1038/bjc.1949.46

**Published:** 1949-09

**Authors:** L. A. Elson, L. F. Lamerton

## Abstract

**Images:**


### THE INFLUENCE OF THE PROTEIN CONTENT OF THE DIET ON THE RESPONSE OF WALKER RAT CARCINOMA 256 TO X-RADIATION.

L. A. ELSON AND L. F. LAMERTON.

*From the Chester Beatty Research Institute, and the Physics Department, The Royal Cancer Hospital, London, S.W.3.*

Received for publication July 16, 1949.

IN an investigation of the body growth- and tumour growth-inhibiting action of carcinogenic substances, it was found that the nature of the inhibition is profoundly influenced by the protein content of the diet (Elson and Warren, 1947 ; Elson and Haddow, 1947 ; Elson, 1949). In rats maintained on a 20 per cent protein diet injection of the carcinogen usually has little immediate effect on body growth although a delayed action, resulting in rapid loss of weight followed by death, may occur later. On the other hand, animals maintained on a 10 per cent protein diet respond to the injection by an immediate retardation of growth.

Treatment with the carcinogenic hydrocarbon 1:2:5:6-dibenzanthracene 24 hours after implantation of Walker carcinoma 256 in rats maintained on a 20 per cent protein diet results in very little difference in size and weight of the tumours from those of controls. If, however, the animals are fed a 5 per cent protein diet the tumours of those similarly treated with dibenzanthracene were found to be only one-quarter the weight of those of controls when the rats were killed 13 days after implantation (Elson and Haddow, 1947). A much greater inhibition of tumour growth has been obtained with derivatives of 4-aminostilbene described by Haddow, Harris, Kon and Roe (1948). Using 2′-chloro-4-dimethyl-aminostilbene, the tumours of the treated animals maintained on a 5 per cent protein diet reached only about 1/20th the size of those of the control animals, whereas the tumours of the treated animals fed on the 20 per cent protein diet did not differ significantly in size from controls (Elson, 1949).

From consideration of these and other results it has been suggested that the growth-inhibiting action of these substances is brought about by an interference with the availability or with the actual synthesis of proteins necessary for growth.

Since nucleic acid metabolism may be closely associated with protein synthesis (Caspersson and Santesson, 1942; Caspersson, 1947; Brachet, 1947), Elson and Harris (1947) investigated the nucleic acids in the liver of rats treated with 1:2:5:6-dibenzanthracene and found an increase in the ratio of pentosenucleic to deoxypentosenucleic acid, caused by a fall in concentration of the latter.

An initial decrease in cellular deoxypentosenucleic acid was found by Stowell (1945) after X-irradiation of transplanted mouse mammary carcinoma, and Mitchell (1942) from investigations of human tumours treated by X-radiation considered that an effect of the radiation was to inhibit the conversion of pentosenucleic to deoxypentosenucleic acid.

It appears therefore that methods by which growth inhibition is achieved by chemicals and by radiation may not be entirely unrelated. Since the inhibition of tumour growth by these chemical substances is profoundly influenced by the protein content of the diet of the animal, it becomes of considerable practical and theoretical importance to study the effect of diet on the response of tumours to treatment by X-radiation. As most of the work on tumour inhibition by these chemical substances has been carried out with the Walker rat carcinoma 256 this tumour has been selected for the present preliminary study of the effect of radiation.

### EXPERIMENTAL.

*Animals.*—The animals used were albino rats of both sexes, weighing between 100 and 150 g. at the time of tumour implantation.

They were kept in individual cages, and maintained either on 20 per cent or on 5 per cent protein diets for at least 7 days before the tumours were implanted. The weight of each rat was recorded at intervals of 2 or 3 days throughout the period of the experiment.

*Diets.*—The 20 per cent protein diet was that described by Elson and Warren (1947, Table I), and the 5 per cent protein diet that described by Elson, Goulden and Warren (1947, Table II). Casein is the main protein constituent of these diets.

### *Radiation technique.*

For the initial experiments on whole body irradiation the rats were placed in small compartments in a thin wooden box. The dosage rate at the centre of the rat's body was determined by measurements in the compartment when partly filled with Spiers' powder (Spiers, 1943). The radiation conditions were: 280 kV., filter 0·5 mm. Cu, half value layer 1·8 mm. Cu, f.s.d. 60 cm. Dosage rate at centre of rat's body, 26 r/min.

For the studies involving the localized irradiation of the tumour, it was necessary first to develop a method whereby the rat could be held firmly in reasonable comfort and the tumour alone irradiated with precision. Anaesthesia was contra-indicated, since it may affect the response of the animal to radiation.

The device eventually developed is shown in Fig. 1. A length of transparent plastic " cloth " is held by means of two clamping bars on an aluminium base. The rat is placed under the plastic material, which is then stretched under the clamping bars. The edge of the plastic cloth near the rat's head must be reinforced with a strip of cellophane one to two inches wide, which serves to hold

FIG. 1.—Photograph of rat in holder designed for X-ray treatment of tumour.

the rat's head down and prevents him biting his way out. The tumour itself projects through a hole in the plastic material, and can be treated by crossfire techniques without irradiation of the rest of the body.

A Victor KX10 plant was used, run at 140 kVp. with a filter of 0·1 mm. Cu, giving radiation of half value layer 0·20 mm. Cu. The applicators used were of f.s.d. 15 cm. Under these conditions the dosage rate at the centre of the tumour is about 140 r/min.

RESULTS.

*Whole body X-radiation.*

The experiments with whole body radiation were designed to approximate to the conditions used in tumour inhibition experiments with chemical substances (Elson and Haddow, 1947). The radiation was commenced 24 hours after tumour implantation, and thereafter daily treatments were given. Two groups of 20 rats were used, one group being maintained on the 20 per cent protein diet and one on the 5 per cent protein diet. All the animals were implanted with Walker Carcinoma 256, and ten animals of each group kept as controls.

In the first experiment a total of 180 r per rat given in 9 daily doses of 20 r, beginning the day after tumour implantation, had no very significant effect on the growth of the tumours in animals maintained on either 20 per cent or 5 per cent protein diets. When the animals were killed 14 days after implanting the tumour the ratio (C/T) of the average weight of the tumours of the control animals (C) to that of the tumours of the treated animals (T) was 1·2 for the 20 per cent protein diet group and 0·9 for the 5 per cent protein diet group.

TABLE I.—*Effect of Whole Body X-Radiation on the Growth of Walker Carcinoma 256 in Rats Maintained on 20 per cent and on 5 per cent Protein Diets.*

100 r given every day for eight days. Total dose = 800 r.
*Radiation conditions* . . . . 280 kV. Filter 0·5 mm. Cu.
Half-value layer 1·8 mm. Cu.

WEIGHT OF TUMOURS AFTER 11 DAYS (*g*.).

| | 20 per cent protein diet. | | 5 per cent protein diet. | |
|---|---|---|---|---|
| | Controls (C). | Treated (T). | Controls (C). | Treated (T). |
| | 23·3 | 6·3 | 17·9 | 7·0 |
| | 29·3 | 15·0 | 21·8 | 2·5 |
| | 21·6 | 6·5 | 9·3 | 4·8 |
| | 27·2 | 12·5 | 25·9 | 2·3 |
| | 27·6 | 5·9 | 24·0 | 4·3 |
| | 29·5 | 8·3 | 29·1 | 1·7 |
| | 30·6 | 12·4 | 23·8 | 3·5 |
| | 15·4 | 2·5 | 11·6 | 2·9 |
| | 17·6 | 10·1 | 23·3 | 3·8 |
| | 28·9 | 6·0 | 22·0 | 4·5 |
| *Mean* | *25·1* | *8·6* | *21·0* | *3·7* |
| | $\frac{C}{T} = 2{\cdot}9$ | | $\frac{C}{T} = 5{\cdot}7$ | |

In a second experiment, carried out in the same manner, the total dose was increased to 800 r, given as 100 r per day for 8 days. This appeared to be close to the maximum tolerated dose. The result is given in Table I. When the animals were killed 11 days after implantation the ratio C/T was 2·9 for the 20 per cent protein diet animals and 5·7 for the 5 per cent protein diet group.

This result is similar to the results observed with chemicals in that a much greater tumour-inhibiting action is obtained in the animals maintained on the low protein diet, but the actual degree of inhibition is not as great as can be achieved by the use of certain chemicals, particularly some of the aminostilbene derivatives (Elson, 1949).

The advantage of X-ray treatment, however, is that it can be applied directly to the tumour in doses much larger than could be tolerated if applied to the whole animal, and the effect of diet under these conditions, which approach more nearly to those used in radiotherapy of human cancer, was next investigated.

*Direct irradiation of established tumours.*

For these experiments the tumours were allowed to grow untreated for 6 days after implantation. Daily estimates of the size of the tumour were then made by measurement with calipers along two axes at right angles. The estimate of the area (sq. mm.) obtained by multiplying these two figures was plotted against the number of days after implantation. A typical curve for the tumour of a control animal maintained on the 5 per cent protein diet is shown in Fig. 2. From the results obtained with more than 20 control animals there appeared to be no very marked difference in rate of growth of the tumour in animals maintained either on 5 per cent or on 20 per cent protein diets. The average size of

FIG. 2.—Typical growth curve of control Walker rat carcinoma 256 in animal maintained on 5 per cent protein diet, with representation of size of tumour 6 days after implantation and at death of the animal.

the tumours 6 days after implantation (when radiation was begun) was very slightly smaller in the 5 per cent protein diet animals (325 mm.$^2$) than in the 20 per cent protein diet group (350 mm.$^2$). In the control animals the average size of the tumours at the death of the animal was also slightly smaller (1900 mm.$^2$) in the 5 per cent protein than in the 20 per cent protein diet animals (2300 mm.$^2$), but the average survival time of the animals of the 5 per cent protein group was slightly shorter (17 days) than that of the 20 per cent protein group (19 days). The rate of growth of the control tumours of the 5 per cent protein group, particularly over the period during which radiation of the treated animals was carried out, was therefore very nearly equal to that of the 20 per cent protein group animals.

*X-radiation*, 250 *r per treatment* (*total* 4000 *r*). (Fig. 3.)

The rats received a dose of 250 r applied direct to the tumour every day except Sundays, a total of sixteen doses being given, the treatment extending over a period of eighteen days. In the group of 4 animals maintained on the 20 per cent protein diet this resulted in a slight retardation of tumour growth compared with that of controls. The average survival time was 28 days, compared with about 19 days for control animals. The group maintained on the 5 per cent protein diet, however, showed considerable inhibition of tumour growth, and the tumours after the first 7 days of treatment ceased to grow and remained fairly constant in size for a considerable time. They then, however,

FIG. 3.—Growth of Walker rat carcinoma 256 treated with X-radiation ; 250 r per day, total dose 4000 r.

——— Rats fed on 20 per cent protein diet.

-·-·-·-·- Rats fed on 5 per cent protein diet.

gradually developed areas of necrosis, which usually become infected, and in some cases the tumour had resumed growth shortly before the death of the animal. The average survival time of the animals (44 days) was considerably longer than that of the high protein diet group. No animal in either group was cured of the tumour by this radiation treatment.

*X-radiation*, 400 *r per treatment* (*total* 4000 *r*). (Fig. 4.)

Since no curative effect on the Walker carcinoma was obtained with a dose of 250 r per day the rate of dosage was increased to 400 r per treatment, the total amount of radiation being still maintained at 4000 r. 400 r was given daily, except Sundays, over a total period of 12 days.

For the first four days after treatment the tumours of the 5 animals comprising the 20 per cent protein diet group grew more rapidly than those of the 5 per cent protein group, and reached an average size of about one and a half times that of the low protein group. After this period the tumours of the 20 per cent protein diet animals began to decrease in size, and eventually two of them regressed completely by a " shelling-out " process and a third remained very small over a long period. The other two, after a considerable period of regression, eventually grew again rapidly and the animals died of the tumour about 45 days after implantation.

In the 5 per cent protein group one tumour regressed completely but the others grew fairly steadily at a much reduced rate for a considerable period, eventually resuming a more rapid growth rate before the death of the animal, which occurred on the average 36 days after implantation.

FIG. 4.—Growth of Walker rat carcinoma 256 treated with X-radiation; 400 r per day, total dose 4000 r.
——·——·—— Rats fed on 20 per cent protein diet.
–·–·–·–·–·– Rats fed on 5 per cent protein diet.

From this result it seems possible therefore that, although the growth-retarding action of radiation may be greater when the animals are maintained on a 5 per cent than on a 20 per cent protein diet, the degree of tumour regression and the ability of the animal to rid itself of the tumour may be favoured by a high protein diet.

This suggests that a better result might be obtained by maintaining the animals on a low protein diet during the period of radiation treatment, so achieving the greatest tumour growth-retarding effect, and then changing to the high protein diet which appears to favour regression of the inhibited tumour and cure of the animal.

This was attempted in another experiment in which the animals in the 5 per cent and 20 per cent protein diet groups were again treated daily except Sundays with 400 r. This time, however, the tumour appeared to be growing rather more rapidly than in the previous experiment, particularly in the 20 per cent protein group. While the behaviour of the tumours of the 5 per cent protein group was very similar to that shown in the first experiment, only slight regression was observed in the 20 per cent protein group. When the regression occurred the size of all these tumours was already greater than the maximum attained by the previous 20 per cent group, and only a slight decrease in size occurred before they again resumed rapid growth. No cures were obtained in animals of either group.

It appeared therefore that regular control of the established Walker tumour could not be effected by a daily dose of 400 r, and in the next experiment the effect of a daily dose of 600 r was studied, together with the effect of changing the diet of the low protein group of animals to the high protein content at the completion of the course of radiation.

*X-radiation,* 600 *r per treatment* (*total* 4000 *r*).

In these experiments a dose of 600 r was given daily on six days, the seventh dose being reduced to 400 r to maintain the total dose at 4000 r. In the first experiment the animals of the 20 per cent protein diet group responded well at first, and nearly all the tumours began to decrease in size after the fourth dose of radiation. Although the tumours then remained small for a considerable time no complete regressions occurred, and all eventually grew again fairly rapidly, leading to the death of the animals.

The tumours of the animals maintained on the 5 per cent protein diet again at first responded to the treatment by growing rather more slowly than those of the 20 per cent protein group. The day following the last radiation treatment the 5 per cent protein group animals were changed over to the 20 per cent protein diet and maintained thereafter on this high protein diet. Under these conditions one tumour "shelled out" 30 days after implantation and one remained very small for more than 50 days, when it eventually regressed completely. The tumours of the other animals of the group remained small for a time, but then grew at about the same rate as those of the 20 per cent protein group animals.

In a repeat experiment a better response was obtained in the group of five animals maintained on the 20 per cent protein diet, and four of the tumours regressed completely. In the five animals of the 5 per cent protein diet group subsequently changed to 20 per cent protein after completion of the radiation treatment three regressions were obtained. The results of these two experiments are thus rather inconclusive.

By giving 600 r per day the total period during which the rats are under radiation treatment is, however, reduced to 8 days. Particularly in view of the poor response obtained in the first experiment with the 20 per cent protein diet group, it seems probable that this period of treatment may not be long enough to secure optimum response. However, to continue with further daily doses of 600 r, thus increasing the total beyond 4000 r, seems contra-indicated by the toxic effects which might arise since in the experiments described above two early deaths occurred not directly attributable to the growth of the tumour.

The best method of treatment of the tumours might therefore be to increase the duration of treatment by fractionation of the doses, giving larger doses early in the treatment, and to reduce the dose as the treatment proceeds and control of the growth of the tumours is being attained.

*X-radiation fractionated doses* (*total* 4000 *r.*). (Fig. 5 and 6.)

For the first experiment with fractionated doses (Fig. 5) the initial dose, given on the sixth day after tumour implantation, was 1000 r. In order to extend the period of treatment, and also to allow the animals to recover from this rather heavy dose, two days were allowed to elapse before the next treatment. Doses of 500 r were given on each of the following two days. No treatment was given on the next day, and then two more doses of 500 r on each of the next two days. After a further two days free from treatment one dose of 500 r was given, and followed after three days' interval by a dose of 250 r, and a final dose of 250 r after another day free from treatment. The total dosage of 4000 r was thus spread over a period of 17 days.

FIG. 5.—Growth of Walker rat carcinoma 256 treated with X-radiation ; extended treatment with initial high dose (1000 r) ; total dose 4000 r.

——•——•—— Rats fed on 20 per cent protein diet.
—•—•—•—•— Rats fed on 5 per cent protein diet.

The growth of the tumours of all five animals maintained on the 20 per cent protein diet was rapidly brought under control and the tumours began to regress. All the animals of this group were eventually cured, complete regression occurring after about 26 days in three animals and after about 43 days in the remaining two.

On the other hand, only one of the five animals receiving similar treatment but maintained on the 5 per cent protein diet was cured. Although growth of the tumours was slowed down considerably four out of the five eventually began to grow again and subsequently caused the death of the animals. Under these conditions therefore the large initial dose of radiation appears to have such a strong inhibiting effect on the tumours that a marked difference is no longer observed between the initial response of low and high protein diet animals. The effect of the high protein diet on the process of tumour regression and on the ability of the animal to rid itself of the tumour now becomes the most important factor in deciding the response of the animal to treatment.

In a repeat of this experiment (Fig. 6) the first dose of radiation, given on the 6th day, was (by an oversight) only 300 r, and this dose was therefore followed by a dose of 1000 r on the 7th day. Doses of 500 r were then given on the 10th, 11th, 13th, 14th and 17th days, and doses of 250 r on the 21st and 24th days. The effect of the preliminary treatment with 300 r may have been to slow down the response of the tumours to the treatment. In the animals maintained on the 20 per cent protein diet regression of the tumours did not commence so early as in the previous experiment with fractionated doses, but again all the animals of this group were ultimately cured, two of the tumours regressing completely just before the 30th day and the other three before the 40th day.

FIG. 6.—Growth of Walker rat carcinoma 256 treated with X-radiation ; extended treatment with initial high dose (1000 r) following dose of 300 r, total dose 4300 r.

———·———·——— Rats fed on 20 per cent protein diet.

—·—·—·—·—·—·— Rats fed on 5 per cent protein diet.

None of the animals of the 5 per cent protein diet group in this experiment was cured of the tumour. The growth of the tumour was considerably retarded in all of them, but eventually rapid growth was resumed, which finally caused death.

In a further experiment reproducing as nearly as possible the conditions of the first fractionated dose experiment four out of the five 20 per cent protein group animals were cured, and only one cure was obtained in the five comprising the 5 per cent protein group.

The net result of the fractionated dose experiments was therefore that of 15 animals maintained on 20 per cent protein diet, 14 cures were obtained, whereas in the corresponding series of 15 animals maintained on the 5 per cent protein diet only 2 cures were effected.

### DISCUSSION.

The effect of nutrition on the response of tumours to radiation appears to be a much-neglected field with regard to experimental animal studies, and indeed also in human cancer therapy. In the experiments reported in this paper, the very definite effect of the protein content of the diet on the response of the animal and the tumour to radiation treatment emphasizes the necessity of a thorough appreciation of the role of nutrition in clinical treatment.

In experiments carried out under the conditions of the " Walker tumour test," as applied to the discovery of growth-inhibitory chemicals by Haddow and his collaborators (Badger, Elson, Haddow, Hewett and Robinson, 1942 ; Haddow, Harris, Kon and Roe, 1948), Elson and Haddow (1947) and Elson (1948) found that the inhibiting action of these chemicals could be largely neutralized by maintaining the animals on a high protein diet. The greatest degree of tumour inhibition was obtained when the animals were maintained on a low protein diet.

It has now been shown that the response of rats implanted with Walker carcinoma 256 to whole body X-radiation under conditions approaching those of the " Walker tumour test " is also greater in those animals maintained on the low protein diet. The protective action of the high protein diet on the inhibition of tumour growth by whole body radiation is, however, not so great as is observed in treatment with some of the chemical inhibitors.

The " Walker tumour test " can be considered as a very efficient sorting test designed to reveal the maximum tumour growth-inhibiting action of the particular substance under test, the compound being usually given by intraperitoneal injection within 24 hours of the implantation of the tumour. Thus the conditions under which this test is carried out are necessarily far removed from those existing in the therapy of human cancer.

It was therefore decided to approach more closely to clinical conditions by allowing the tumour to become well established, and to grow to a definite size before treatment was commenced, the response of the tumour being followed by daily measurements of tumour size. This technique has been adopted in the present studies of the localized irradiation of tumours, and it is hoped that its use will also eventually allow a direct comparison to be made between the action of radiation and that of chemicals suitably selected by the sorting test, and make possible a study of the combined effect of chemotherapy and radiotherapy.

The experiments on the localized irradiation of established tumours reveal a greater inhibitory action in the animals maintained on the low protein diet than in those maintained on a high protein diet, the difference in response being most marked when the radiation is applied in frequent small doses (250 r daily, total dose 4000 r, Fig. 3), although this form of treatment rarely leads to complete regression of the tumour. However, if the same total dose is applied in the form of larger daily doses, particularly if an initial dose of 1000 r is given, with extended time intervals between successive smaller doses (Fig. 5 and 6), regression of many of the tumours can be achieved. The number of regressions and cures is then found to be much greater in the animals maintained on the high protein diet.

It is clear therefore that in considering the response to radiation of the tumour, one must separate to some extent the processes which determine—

(*a*) The initial response of the tumour.

(*b*) The elimination of the inhibited tumour and cure of the animal.

Process (*a*) is found to be favoured by a low protein diet, and process (*b*) by a high protein diet.

In work on the inhibition of tumours by chemicals, the first process, (*a*), has, in general, been the one observed, and it is now seen that the effect of diet on this process is similar for both X-radiation and chemical treatment. With X-ray treatment, however, it is possible to apply direct to the tumour a sufficiently large dose of radiation to bring about an adequate initial growth-inhibitory response, even in animals maintained on a high protein diet, so that the subsequent outcome of the treatment then depends almost entirely on process (*b*), and a favourable result is thus to a very large extent dependent on a high protein content of the diet.

Though the results obtained may be related to some extent to the particular characteristics of the tumour used, it is very interesting to note how closely the two processes distinguished in the radiation response of the tumour follow the concepts of " intra-cellular response " and " inter-cellular response " put forward by Koller and Smithers (1946) from studies in clinical radiotherapy. Koller and Smithers suggest that ". . . Tumour regression as the result of irradiation is initiated by an intra-cellular response, but is carried on by an inter-cellular response which follows and plays an important part in the success of any treatment. The intra-cellular reaction is directly responsible for the death of individual cells, and its rate is of particular importance at the beginning of treatment. The inter-cellular reaction is manifested through the repair processes, and depends on the construction of the tumour tissue as a whole. This reaction is conditioned and influenced by the intra-cellular response ; in this sense it is an indirect effect of radiation . . ."

It seems likely that this " inter-cellular " response may constitute an important part of what we have described as process (*b*), and have shown to be favourably influenced by a high protein diet. If the " inter-cellular response " is related to the repair processes, then it is very reasonable to suppose that nutritional factors can affect the elimination of the tumour in radiation treatment, and indeed proteins would be of special importance in view of their known value in the healing of burns and wounds.

Clearly the present work can only be regarded as a preliminary study. A number of different lines of investigation have now to be followed, including the question of whether a certain type of protein or any of its constituent amino-acids are particularly concerned, and also whether other nutritional factors can contribute to the effectiveness of the treatment. Similar investigations with other animal tumours must also be carried out. For the fuller appreciation of these results a systematic histological and cytological study of the tumour at various stages of the radiation treatment is essential, and this study is now under way.

### SUMMARY.

The effect of diets containing 20 per cent and 5 per cent protein respectively on the response of Walker rat carcinoma 256 to whole body X-radiation and to localized irradiation of the tumour has been investigated.

For whole body radiation (100 r daily for 8 days commencing 24 hours after tumour implantation) or for localized irradiation of the established tumour with 4000 r total dose applied in daily doses of 250 r, commencing 6 days after tumour implantation, inhibition of tumour growth was greatest in those animals maintained on the low protein diet, but no complete regressions of the tumour were obtained.

With localized irradiation of the tumour, when the total dose of 4000 r was applied in larger daily doses, complete regression of some tumours was observed, and the most favourable results were obtained by application of an initial dose of 1000 r with extended time intervals between successive smaller doses. Complete regression of the tumours was then obtained in about 90 per cent of the animals maintained on the high protein diet, but in only about 15 per cent of these maintained on the low protein diet.

It is suggested that two effects are concerned in the response of tumours to radiation, (*a*) the initial inhibition of tumour growth, (*b*) the elimination of the inhibited tumour and cure of the animal. Process (*a*) is favoured by a low protein diet, and process (*b*) by a high protein diet.

We would like to express our appreciation of the interest taken in this work and the encouragement given by Professor A. Haddow and Professor W. V. Mayneord, and of the able assistance of Miss C. B. Barrett, Miss M. Winsborough and Mr. R. Poppy. We also wish to express our thanks for grants supporting this investigation from the British Empire Cancer Campaign, the Anna Fuller Fund, the Jane Coffin Childs Memorial Fund for Medical Research and the U.S. Public Health Service; also for facilities provided by Imperial Chemical Industries Limited.

### REFERENCES.

Badger, G. M., Elson, L. A., Haddow, A., Hewitt, C. L., and Robinson, A. M.—(1942) *Proc. Roy. Soc.*, B, **130**, 255.

Brachet, J.—(1947) *Cold Spring Harbor Symposia on Quantitative Biology*, **12**, 18.

Caspersson, T.—(1947) *Symp. Soc. exp. Biol.*, **1**, 127.

*Idem* and Santesson, L.—(1942) *Acta radiol. Stockh. Suppl.*, 46.

Elson, L. A.—(1948) *Acta Union int. Cancer*, **4**, 396.—(1949) *Symp. Soc. exp. Biol.*, **3**, 327.

*Idem* and Haddow, A.—(1947) *Brit. J. Cancer*, **1**, 97.

*Idem* and Harris, R. J. C.—(1947) *Ibid.*, **1**, 327.

*Idem* and Warren, F. L.—(1947) *Ibid.*, **1**, 86.

*Idem*, Goulden, F., and Warren, F. L.—(1947) *Ibid.*, **1**, 80.

Haddow, A., Harris, R. J. C., Kon, G. A. R., and Roe, E. M. F.—(1948) *Phil. Trans.*, A, **241**, 147.

Koller, P. C., and Smithers, D. W.—(1946) *Brit. J. Radiol.*, **29**, 89.

Mitchell, J. S.—(1942) *Brit. J. exp. Path.*, **23**, 296, 309.

Spiers, F. W.—(1943) *Brit. J. Radiol.*, **16**, 90.

Stowell, R. E.—(1945) *Cancer Res.*, **5**, 169.